# Complete mitochondrial genome of *Ciconia nigra* (Ciconiiformes: Ciconiidae)

**DOI:** 10.1080/23802359.2017.1310608

**Published:** 2017-04-24

**Authors:** Mu-Yeong Lee, Hey Sook Jeon, Young-Jun Kim, Junghwa An

**Affiliations:** a Animal Resources Division, National Institute of Biological Resources, Incheon, Republic of Korea;; b Department of Veterinary Medicine, National Institute of Ecology, Seocheon, Chungcheongnam-do, Republic of Korea

**Keywords:** *Ciconia nigra*, mitogenome, mitochondrion, Black Stork

## Abstract

The complete mitogenome of the Black Stork *Ciconia nigra*, obtained using a PCR-based method, is reported. It is 17,905 bp, slightly A + T biased (30.8% A, 31.5% C, 14.1% G, and 23.6% T), and comprises 13 protein-coding genes, 2 ribosomal RNA genes, 22 transfer RNA genes, and 1 putative non-coding gene. The two kinds of tandem repeat units found in D-loop (2316 bp) generated a length polymorphism between this and the previously reported D-Loop of *C. nigra* from China (2206 bp). A frameshift mutation was observed in *ND3*. The phylogenetic analysis clustered *C*. *nigra* with other *Ciconia* species.

The Black Stork, *Ciconia nigra*, a large wading Ciconiidae, has a wide geographical range and breeds throughout the Palearctic, from Malawi and Namibia to South Africa (Elliott et al. 2017). In South Korea, a very limited number of individuals have been observed during winter (NIBR 2011). Thus, *C nigra* is classified as an Endangered species II by the Ministry of Environment of Korea, and designated a Natural Monument (No. 200) by the Cultural Heritage Administration of Korea (CHA). Here, we determined the mitogenome sequence of *C. nigra* from South Korea and compared it with the previously released one.

Following the CHA regulations, a blood sample (IN749) was collected from a rescued individual at the National Institute of Ecology and deposited in the National Institute of Biological Resources at Incheon, South Korea. Total genomic DNA was extracted from this sample using the DNeasy Blood & Tissue Kit (Qiagen, Valencia, CA) according to the manufacturer’s instructions. The mitogenome was amplified from eight overlapping fragments and determined using the primer-walking method. Fragments were assembled in Geneious Pro 8.1.9 (Biomatters; Kearse et al. 2012) and mitogenome annotation was performed in DOGMA (Wyman et al. 2004) and ARWEN (Laslett & Canbäck 2008).

The complete mitogenome of *C*. *nigra* (GenBank No. KY767670) was 17,905 bp in length and contained 13 protein-coding genes, 2 ribosomal RNA genes, 22 transfer RNA genes, and a putative non-coding gene (D-loop). The arrangement of genes was that typically found in vertebrate mitogenomes. Average base composition was 30.8% A, 31.5% C, 14.1% G, and 23.6% T, with a slight AT bias (54.4%). The D-loop sequence found here (2316 bp) was longer than that of mitogenome KF906246 deposited in GenBank (2206 bp), due to the two kinds of tandem repeats found in the D-loop of *C. nigra* from South Korea. The other protein-coding genes of the two Black Stork mitogenomes were identical. In *ND3*, a frame shift mutation was observed, which was also reported in the mitogenome of Black Stork from China (Liu et al. 2016) and in some birds and turtles (Mindell et al. 1998).

The concatenated sequences of the 13 protein-coding genes (11,408 bp) from eight closely related species were used to evaluate phylogenetic relationships within the family Ciconiidae and Threskiornithidae. The neighbour-joining tree constructed in MEGA 6.0 (Tamura et al. 2013) showed *C*. *nigra* within the *Ciconia* spp. clade, as expected (Figure 1). The data generated in this study will help exploring the genetic diversity of the Endangered *C. nigra* and contribute to its molecular identification.

**Figure 1.**
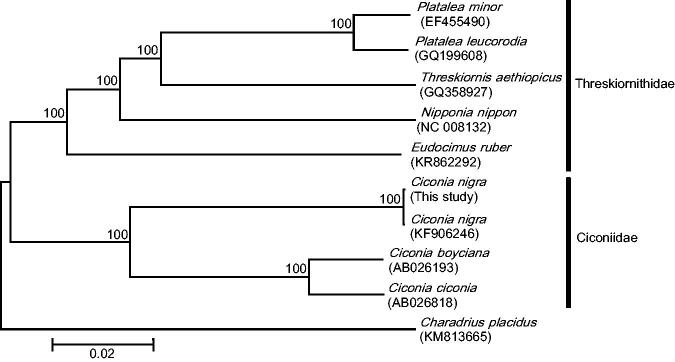
Neighbour-joining phylogenetic tree constructed for eight species of Threskiornithidae and Ciconiidae, based on the concatenated nucleotide sequences of the 13 protein-coding genes found in their mitogenomes. Numbers above each node indicate bootstrap support values. GenBank accession numbers are indicated next to species designations.
